# Child Sexual Abuse: Forensic Medical Assessment of the Traumatic Injuries Over the Victim’s Body

**DOI:** 10.7759/cureus.49873

**Published:** 2023-12-03

**Authors:** Biliana Mileva, Metodi Goshev, Mihaela Georgieva, Ilina Braynova, Alexandar Alexandrov

**Affiliations:** 1 Department of Forensic Medicine and Deontology, Medical University Sofia, Sofia, BGR

**Keywords:** child maltreatment, injury pattern, clinical forensic medicine, child abuse, sexual assault, sexual violence, sexual abuse trauma, child sexual abuse

## Abstract

Child sexual abuse is a public health problem that affects children worldwide in all ethnic, educational, and socioeconomic groups. These assaults are extremely dangerous not only due to their direct physical traumas received at the time of the abuse, but they also have long-term consequences that can worsen the future quality of the victim’s life. A retrospective study of all cases related to child sexual abuse for five years was performed by materials of the Clinic of Forensic Medicine and Deontology, Sofia, Bulgaria. Ninety-five cases involve children from both genders. The traumatic injuries were carefully examined and classified according to their localization over the victim’s bodies and based on the time that had passed after the reported assaults. In cases of sexual violence, the most informative and pointing at the exact type of violence are the injuries situated in the anogenital area. Too often, there is a lack of physical findings, depending on the type of sexual violence or associated with the prolonged time that passes after the crime. Children are unaware of what they have to do after suffering such traumas, or they are scared to share their experience with different family members, which can lead to late forensic examination and lack of physical and biological findings, which are the most critical traces in the criminal prosecution of the crimes and this can be a possibility for the perpetrator not to be charged for his unlawful actions.

## Introduction

“Child abuse syndrome” and “Child maltreatment” are known terms associated with different forms of child abuse and are well-recognized worldwide. They include physical, sexual, and emotional violence, or a combination [1,2]. Each form of malicious act against minors is dangerous since it can have direct effects associated with the type of sustained injuries over the victim’s body or late effects, which can have unpredictable consequences for the victims and their families - worsening their mental, physical, and emotional well-being [1-6]. A significant aspect of the problem is the fact that there is a substantial lack of correspondence between the actual rates and the number of cases reported to the authorities [1,7]. It is well known that most of the assaults against children are not reported [7], or if registered, this is done too late when there are no prominent physical findings left. The last leads to a lack of proof and gives the perpetrator of the crime a chance to get out of the situation as an innocent person.

The current study was presented as an abstract and poster presentation in 2020 at the ХХХ Anniversary International Scientific online meeting of the Union of Scientists - Stara Zagora, Bulgaria.

## Materials and methods

This study aimed to perform a retrospective analysis of all the cases related to child sexual abuse over five years - from January 1, 2015, to December 31, 2019, by materials from the Clinic of Forensic Medicine and Deontology, Sofia, Bulgaria. Reports concerning sexual violence were carefully examined. For the studied period, a total of 261 cases were found concerning cases of sexual violence against women and minors. Since the aim of the study was to examine the injury pattern in cases of sexual violence against children, the number of assaulted women (aged above 18) was excluded. For each reported case concerning an allegation of sexual violence, the following information was collected - the victim's gender, time that has elapsed following the assault, and a detailed description of the sustained traumatic injuries in the anogenital area and in other parts of the human body. The results were presented in tables concerning information for traumatic injuries, specifically in the anogenital region; out of them, cases where there was a combination of injuries in the anogenital area and other parts of the body, were shown separately (with a description of the type of the sustained injury - bruise, abrasion, laceration, and localization). The tables also include information for the group of injured children with injuries to different sites of the body but without injuries to the anogenital region and a number of children who did not have any visible traumas at the time of the examination.

The examinations were performed by the forensic pathologist who was on duty at the clinic. It includes interviews with the victim and family members, gathering information from the police (when such is available), detailed physical examination, and collection of samples (swabs from various areas of the body, and especially from the anogenital area, which have been handed over to the investigating authorities for further examinations and analyses when such are needed).

Data was collected anonymously and is presented as absolute values.

## Results

For the studied period, 10139 forensic medical examinations were performed at the Clinic of Forensic Medicine and Deontology in Sofia, Bulgaria. Out of them, a total of 95 were cases reported as sexual assaults against minors. The gender distribution of the cases showed that 83 of the victims were girls, and 12 were abused boys.

The first step of our study was to divide all the reported cases of sexual abuse against minors by the time passed after the assault. The results showed that we have two peaks. The first one shows that the most reported are the cases during the first 24 hours after the abuse, and the second one shows a significant number of reported cases three weeks or longer following the assault. The results are almost equal - in the first 24 hours, we have 29 reports of assaults against girls and four cases concerning crimes against boys. Three or more weeks following the assault, we have a total of 34 reported cases, 29 associated with violence against females. During the second day, six cases were reported, with five injured girls and one injured boy. On the third day following the attack, seven reports were made. Six of them were with data on abuse against girls. Four days following sexual violence, there were no reported cases of boys, but there were five cases with girls involved. On the fifth day, there were no reported cases. On the sixth day, only three reports with information on sexual crime against girls were found. On the next - seventh day, there was only one reported case, again against a girl. Two weeks following sexual abuse, according to our protocols, there were a total of four cases, three of them being girls and one boy. Three weeks following the crime, there were only two reported cases of girls. After this period - three weeks or more following a possible sexual abuse against minors, there were a total of 34 forensic medical examinations, with 29 of them associated with violence against girls and five cases of sexually abused boys.

**Figure 1:**
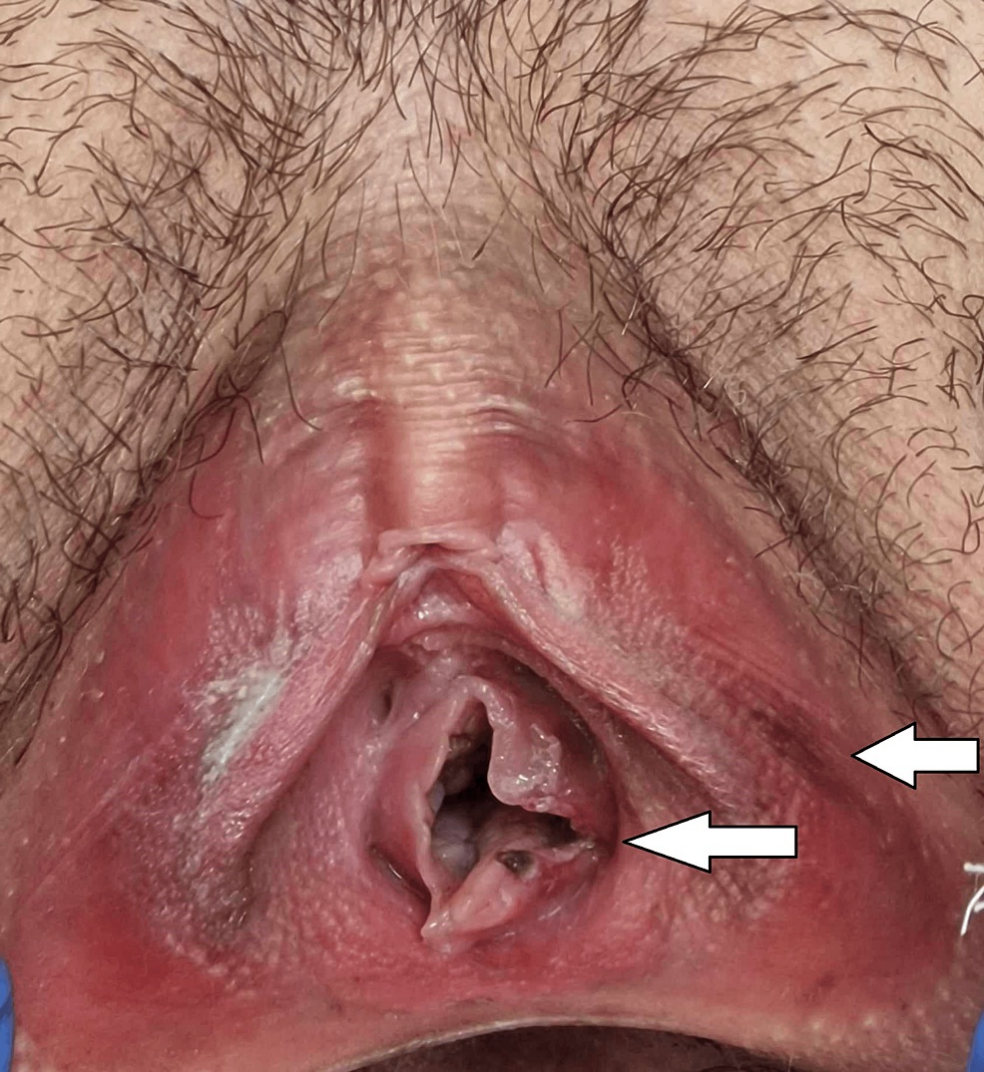
Young girl with fresh hymenal laceration and bruises on the labia

The second aspect of our study was to examine all the cases and make a forensic assessment of the sustained traumatic injuries by the time passed after the assault for both genders. As mentioned earlier, during the first 24 hours, we had a total of 32 examinations - 29 concerning cases of insulted girls and three cases of reported maltreatment against boys. The results are shown in Table 1 and Table 2 for both genders. It is well seen that for the girls, out of these 29 forensic medical examinations, only in 13 cases, there is clear evidence of penetration injury - including hymenal lacerations, with bruised and bleeding margins, perianal lacerations in combination with injuries to the labia (Figure 1).

**Table 1:** Distribution of the traumatic injuries over the victim’s body in correlation with the time passed after the assault – sexually abused girls

	Injuries in the anogenital area	Combination of injuries – anogenital area and other parts of the body (from the group of cases with data for anogenital injuries)	Traumatic injuries to other parts of the body (cases without visible signs of anogenital injuries)	Without traumatic injuries associated with the type of sexual violence (oral sex, frottage, threat) and with the presence of old and already healed hymenal ruptures
Day 1	13 cases – fresh hymenal lacerations, with bruised and bleeding margins; perianal lacerations; abrasions and bruises of the labia and vaginal vestibule.	5 cases – bruises and abrasions on the face, neck, upper limbs, thighs, and buttock area.	6 cases – mostly concentrated in the neck area, armpit, and thigh – linear or scratch abrasions and bruises.	10 cases
Day 2	1 case – fresh laceration of the posterior fourchette, abrasion on the hymen	-	1 case – abrasions and bruises on the armpits and thighs.	3 cases
Day 3	1 case – fresh laceration of the hymen with swollen and reddened margins	1 case – linear abrasions on the limbs	1 case – bruises on the head and thighs	4 cases
Day 4	-	-	1 case – abrasions and bruises on the neck; abrasions of the back and thighs	4 cases
Day 5	-	-	-	-
Day 6	1 case – hymenal laceration with slightly bruised margins	-	2 cases – bruises and abrasions on the thighs, and abrasions on the face and neck.	-
Later	-	-	-	35 cases

**Table 2:** Distribution of the traumatic injuries over the victim’s body in correlation with the time passed after the assault – sexually abused boys

	Injuries in the anogenital area	Combination of injuries – anogenital area and other parts of the body (from the group of cases with data for anogenital injuries)	Traumatic injuries to other parts of the body (cases without visible signs of anogenital injuries)	Without traumatic injuries associated with oral sex, frottage, threat, or the time elapsed following the assault
Day 1	2 cases – bruises and lacerations on the perianal region; staining with feces	-	-	2 cases
Day 2	1 case – bruises on the perianal area, staining with feces	1 case – linear abrasions around the neck and thighs	-	-
Day 3	1 case – perianal lacerations	-	-	-
Day 4	-	-	-	-
Day 5	-	-	-	-
Day 6	-	-	-	-
Later	-	-	-	6 cases

**Figure 2:**
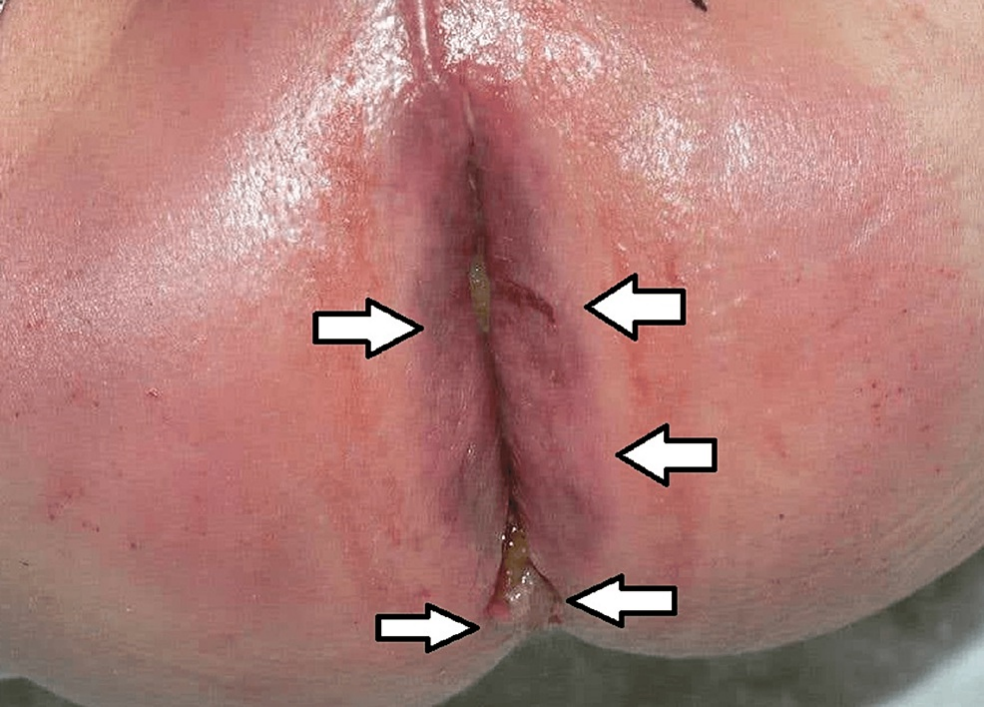
Young boy with perianal bruises, lacerations and staining with feces.

In the five cases mentioned above, there was a combination of injuries in different anatomical regions - in the anogenital area and other body parts. In six cases, there was no clear evidence or even suspicion of sexual abuse, which means that there were no injuries in the anogenital area. Still, there were injuries to other parts of the body - presented as linear/scratch abrasions and bruises in the region of the neck, armpits, and thighs. In 10 cases, there were no traumas over the victim's body. For the boys, we have found four reported cases on the first day following the assault - in two of them, there was clear evidence of penetration associated with fresh bruises and lacerations of the perianal region (Figure 2), and in the other two cases, no traumatic injuries were found over the victim's bodies.

The distribution of the injuries for the following days is shown in the tables. The last day on which we still have clear evidence for penetration injury, according to our protocols, hymenal laceration with slightly bruised margins, is the sixth day following the assault. After this period, there were no visible signs of physical and sexual abuse. The only visible findings found in some of the cases after this period were just old and already healed hymenal ruptures. The last cannot be directly associated with sexual abuse, especially in girls who reported being sexually active before the assault.

The results for the boys are similar - half of the reported cases are reported acutely - during the first three days after the abuse, with still visible findings over the body.

## Discussion

As discussed in the previous study, there is a significant prevalence of female victims of child sexual abuse over boys [8]. The last has been confirmed in different surveys [9-10]. Working with injured children is a delicate act and must be performed in a specific manner to avoid further mental injury to the child [11]. The doctor should find the most suitable approach depending on the situation to understand what has happened - since the minors, especially the youngest of them, are unaware of the different acts of sexual violence. Moreover, they could think that they have done something wrong, so they could be scared to talk, or they could be ashamed. Understanding the situation based on the child’s allegation is crucial, but of utmost importance in cases of child sexual abuse is obtaining physical evidence from the crime [12,13]. The examination should be performed acutely, if possible, immediately after the crime, since if done later or non-acutely, most of the abused children will not have signs supporting the crime - injuries to the anogenital region [14].

Moreover, even without traumatic injuries, when examined acutely, material for further DNA analysis can be taken to confirm the possible crime. It is well known that DNA is predominantly recovered when the examination is conducted less than 24 hours from the time of the assault - the so-called “24-hour rule” [15]. The parents must observe the attitude of their children, and if they notice something different, they have to try to understand what was the cause of this behavioral change. If they feel not competent enough to do it, they have to ask others for professional help. The effects of a malicious act on children depend not only on the type of crime but also on the child’s personality and emotional state. This is why the approach and, later, the treatment of sexually abused children is complex and individual for each child and family [16].

According to our survey, there are two peaks in the cases of sexual abuse against children, based on the time that has passed after the assaults. The first group was examined acutely - immediately after the accident - with the presence of physical findings that support the crime in most cases. The second group is the ones who were examined too late - weeks after the assault, which makes it extremely difficult to prove it. In most of the reported cases, the children were scared to talk about the abuse because they were threatened somehow by the perpetrator. Eventually, the parents understand what has happened and come for an examination, but there are no physical findings to support the child's allegations. Lack of physical findings as traumatic injuries over the victim’s body could be explained not only by the delay of the examination, but by the type of sexual violence - it is well known that some acts do not include physical contact like voyeurism, exhibitionism, or different type of acts with physical contact, but without penetration like oral sex, interfemoral intercourse, heavy petting, and frottage. The act can include penetration - fingers, or other objects, genital or anal intercourse [17]. Based on examination of sexually abused children, authors have divided the physical findings into three main groups - suspicious for abuse - including immediate anal dilatation, extensive venous congestion, distorted irregular anal folds, abrasions or lacerations in the vestibule or on the labia, or perianal lacerations. Findings suggestive of abuse/penetrations are a combination of two or more suspicious anal findings or more suspicious genital findings; scar or fresh laceration of the posterior fourchette with sparing on the hymen, scar in the perianal area. Clear evidence of penetration injury is an area with an absence of hymenal tissue, which is confirmed in the knee-chest position, hymenal lacerations, perianal laceration of posterior fourchette, extending to involve hymen [18]. Supportive findings for sexual abuse, according to other researchers, are contusions of the skin and the mucosa in the genital or anal area, with ruptures of the genital muscles and tissues, and the late consequences - sexually transmitted infections or even pregnancy [19]. Our study confirms those mentioned above as most common; the findings include fresh hymenal lacerations with bleeding and bruising of the margins, perianal lacerations, abrasions and bruises of the labia and vaginal vestibule, and lacerations of the posterior fourchette. The most common localization of traumatic injuries outside of the anogenital area, according to our study, is neck, armpits, and thigh - including linear or scratch abrasions and contusions. Signs of relatively “fresh” penetration were found on the sixth day following the abuse and were presented as hymenal tearing with slightly bruised margins.

The most significant limitation of the current study is the lack of information concerning the results from the samples taken for the additional examinations (swabs from different parts of the victim's body for further DNA analyses), the results which could additionally confirm or exclude possible sexual intercourse.

## Conclusions

The examination of children, victims of sexual abuse, the interpretation of the sustained injuries or the lack of injuries, and the diagnosis must be made with special care and professional knowledge. The wrong interpretation of the findings associated with the case can have severe and long-term consequences for the injured child and his future life.
